# Delayed primary anastomosis for repair of long-gap esophageal atresia: technique revisited

**DOI:** 10.1007/s00383-022-05317-6

**Published:** 2022-12-08

**Authors:** Florian Friedmacher

**Affiliations:** Department of Pediatric Surgery, University Hospital Frankfurt, Goethe University Frankfurt, Theodor-Stern-Kai 7, 60590 Frankfurt, Germany

**Keywords:** Esophageal atresia, Tracheoesophageal fistula, Long gap, Delayed primary repair, Anastomosis, Outcome

## Abstract

The operative management of patients born with long-gap esophageal atresia (LGEA) remains a major challenge for most pediatric surgeons, due to the rarity and complex nature of this malformation. In LGEA, the distance between the proximal and distal esophageal end is too wide, making a primary anastomosis often impossible. Still, every effort should be made to preserve the native esophagus as no other conduit can replace its function in transporting food from the oral cavity to the stomach satisfactorily. In 1981, Puri et al. observed that in newborns with LGEA spontaneous growth and hypertrophy of the two segments occur at a rate faster than overall somatic growth in the absence of any form of mechanical stretching, traction or bouginage. They further noted that maximal natural growth arises in the first 8–12 weeks of life, stimulated by the swallowing reflex and reflux of gastric contents into the lower esophageal pouch. Since then, creation of an initial gastrostomy and continuous suction of the upper esophageal pouch followed by delayed primary anastomosis at approximately 3 months of age has been widely accepted as the preferred treatment option in most LGEA cases, generally providing good functional results. The current article offers a comprehensive update on the various aspects and challenges of this technique including initial preoperative management and subsequent gap assessment, while also discussing potential postoperative complications and long-term outcome.

## Introduction

Esophageal atresia encompasses a spectrum of complex congenital abnormalities of the esophagus, most likely resulting from embryological disruptions during normal foregut separation [1–4]. Its global prevalence, established from national and international registries, currently ranges between 1.27 and 4.55 per 10,000 births [5, 6]. Pure esophageal atresia without tracheoesophageal fistula is a rarer type of malformation, occurring in approximately 8% of neonates born with esophageal atresia and an estimated prevalence of 1 in 40,000 births [7]. Still, there remains a lack of consensus on the precise definition and determination of long-gap esophageal atresia (LGEA) [8–12]. While many discussions have focused mainly on pure esophageal atresia [13, 14], the majority of LGEA cases actually present in association with a distal tracheoesophageal fistula [15].

Despite significant improvements in prenatal diagnosis, most patients with esophageal atresia continue to be diagnosed after birth [16, 17]. Nevertheless, it remains a life-threatening condition, and without surgical intervention, it is not compatible with life. William Ladd reported the first survivors of a staged operative treatment with primary gastrostomy and delayed esophageal reconstruction in 1939 [18, 19]. The first successful primary repair of esophageal atresia was performed by Cameron Haight in 1941 [20, 21]. Subsequently, survival post reconstructive surgery has increased from universally fatal to almost 95% [22, 23], which is likely due to advances in neonatal intensive care and anesthesia, refined surgical techniques, parenteral nutrition and antibiotics [24, 25]. Nowadays, even newborns with very low birth weight and severe cardiac defects survive [26]. However, the high prevalence of prematurity, coexisting anomalies and LGEA frequently complicate the care of these patients and may prevent an immediate primary repair [27, 28]. Thus, operative management of neonates with LGEA still represents a major challenge for most pediatric surgeons, in particular when primary end-to-end anastomosis is not achievable [29–31]. The surgical approach is generally determined by the distance between the proximal and distal esophageal segment and the presence of a tracheoesophageal fistula. Normally, every effort should be made to preserve the native esophagus as no other conduit can replace its function in transporting food from the oral cavity to the stomach satisfactorily [32]. During the past decades, there have been substantial developments in the operative concepts of LGEA repair, and it is widely recognized today that delayed primary anastomosis of the esophagus is not only attainable but also the preferred treatment option in most cases [33]. This article offers a comprehensive update on the various aspects and challenges of this technique including initial preoperative management and subsequent gap assessment, while also discussing potential postoperative complications and long-term results.

### Historical background

Over the years, various surgical approaches have been suggested to decrease the distance between the two esophageal ends in LGEA to achieve a delayed primary anastomosis. In 1965, Howard and Meyers were the initial advocates of periodic manual bougienage of the upper esophageal segment, resulting in elongation of the pouch, followed by a subsequent primary anastomosis 5–8 weeks later [34]. Bougienage of the distal esophageal pouch in addition to the proximal pouch was first published by Lafer and Boley in 1966 [35]. Rehbein and Schweder used a temporary silver prosthesis to create a fistula between the two esophageal segments [36]. In 1972, Thomasson described an elongation of the upper esophageal pouch by mercury-filled bags, and Livaditis et al. introduced circular myotomy of the esophagus to enable a primary anastomosis [37, 38]. The use of silk sutures for esophageal auto-anastomosis by producing a mucosa-lined fistula was proposed by Shafer and David in 1974 [39]. Hendren and Hale reported their experience with electromagnetic stretching of the two esophageal segments in 1975 [40]. In 1980, Gough detailed the fashioning of an anterior full-thickness flap when opening the proximal blind-ending pouch, in this way allowing a wider gap to be bridged [41].

In 1981, Puri et al. published their experience in six newborns with pure esophageal atresia that spontaneous growth and hypertrophy of the two blind-ending esophageal ends occur at a rate faster than overall somatic growth in the absence of any form of mechanical stretching, traction or bouginage [42]. The maximal natural growth of the two esophageal segments occurs in the first 8–12 weeks of life [42]. The stimuli to such natural growth are the swallowing reflex and the reflux of gastric contents into the lower esophageal pouch [43]. Since then, Puri et al. recommended initial gastrostomy placement and continuous suction of the upper esophageal pouch, followed by delayed primary anastomosis as the ideal procedure for the management of newborns with LGEA [42, 43]. In 1994, Boyle et al. confirmed that even in cases of ultralong-gap esophageal atresia (i.e., more than 3.5 cm), a delayed primary anastomosis is feasible [44].

### Initial preoperative management and subsequent gap assessment

**Fig. 1:**
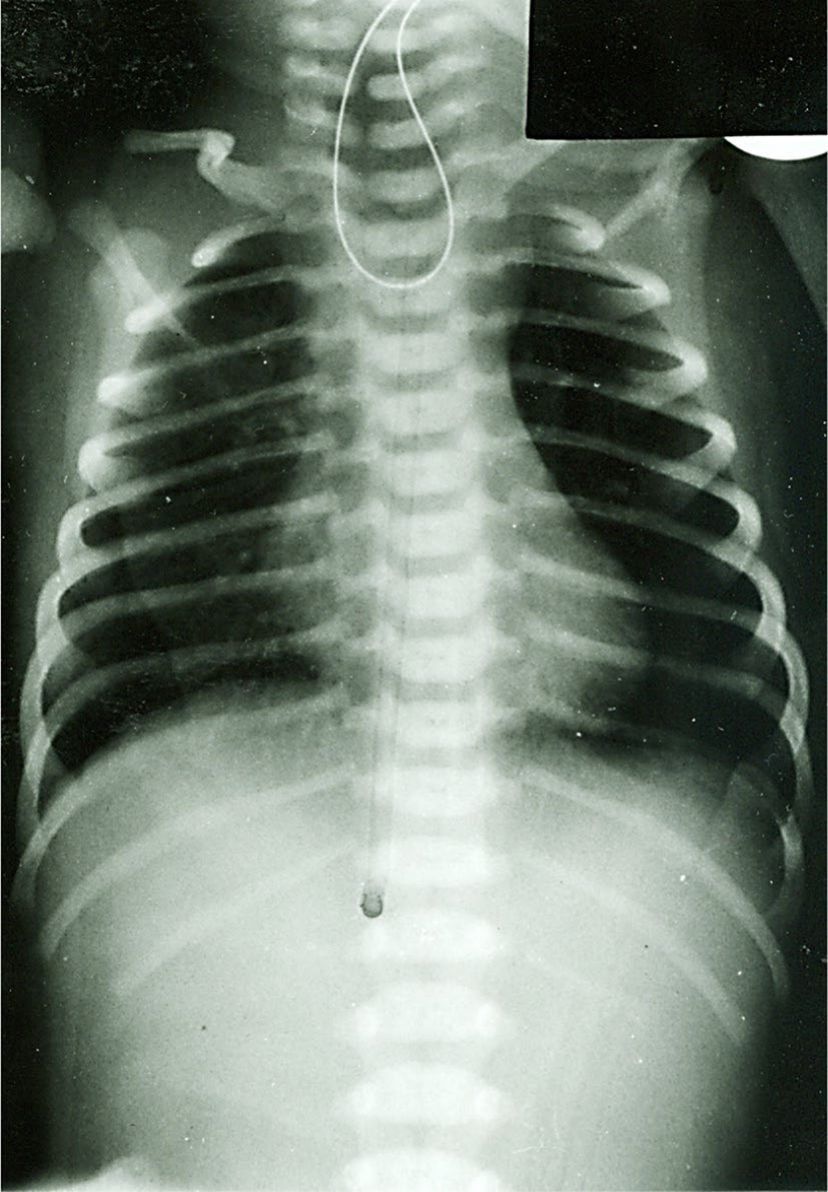
Plain X-ray of a newborn with LGEA showing the radiopaque gastric tube in the blind-ending upper esophageal pouch and no air below the diaphragm (“gasless abdomen”)

Following initial stabilization, normal care and monitoring of the newborn with esophageal atresia are initiated. An 8 Fr or 10 Fr double-lumen Replogle tube should be inserted through the nostril into the upper esophageal pouch and kept under continuous suction to prevent aspiration of saliva and choking episodes. LGEA can be suspected based on a plain X-ray not showing any intra-abdominal air (i.e., Gross type A or B) (Fig. 1) or the fact that primary anastomosis is not achievable in cases with esophageal atresia Gross type C or D, due to a gap length of more than 2–3 cm or 3–4 vertebral bodies [45]. To establish early enteral feeding and ensure adequate nutritional support, a gastrostomy is created on the first or second day of life. One should also look for any coexisting anomalies that may potentially influence the further management (e.g., VACTERL association, CHARGE syndrome and major chromosomal aberrations). With pure LGEA, the patient is maintained supine in Trendelenburg position for efficient suction of the upper esophageal pouch and to allow reflux of gastric contents into the lower esophageal segment. In the presence of a distal tracheoesophageal fistula, the patient should be placed in reverse Trendelenburg position with the head up to minimize reflux of gastric contents into the trachea. The gastrostomy is kept closed between feedings to encourage gastroesophageal reflux and distension of the lower esophageal end. An important factor stimulating hypertrophy of the lower esophageal pouch is the reflux of gastric contents owing to gastroesophageal junction incompetence of newborns and young infants.

**Fig. 2:**
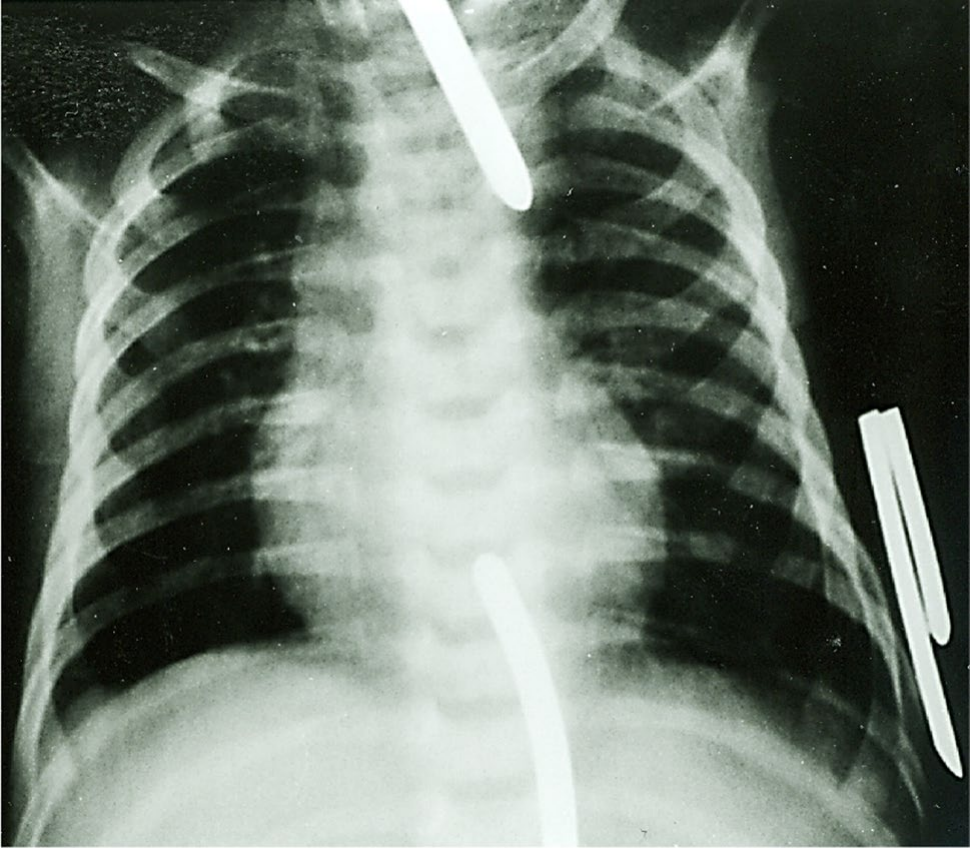
First measurement of the gap between the upper and lower esophageal segment by using radiopaque bougies at 2 weeks of age. The gap is approximately five vertebral bodies long. Care must be taken to not exert excessive pressure on the metal bougies

Various techniques have been described to monitor the elongation of the two esophageal segments and to assess the intermediate gap length, respectively. Injection of water-soluble contrast via the gastrostomy tube may be the simplest and oldest method to evaluate the lower esophageal pouch. Most authors favor radiological assessment of the interceding gap by insertion of metal bougies (e.g., Hegar dilators or urethral sounds) in the upper and lower esophageal segment [46, 47]. Alternatively, the anesthetist is asked to pass a radiopaque tipped catheter into the upper pouch, while the surgeon introduces a rigid metal bougie through the gastrostomy into the lower esophagus. The distance between the two ends can be quantified either by laying the patient on a ruler with radiopaque markings or by counting the number of vertebral bodies between the two segments (i.e., one vertebral body is equal to approximately 1 cm). It is vital not to apply too much pressure to the bougies while measuring the gap between the ends as this can result in errors in estimation of the distance between the two segments [48]. Caffarena et al. [49] first reported inserting an endoscope in both the upper and lower esophageal pouches for measurement of LGEA. Chan and Saing [50] combined flexible endoscopy and fluoroscopy in the assessment of the gap between the two esophageal pouches. Gross et al. [51] demonstrated how fiberoptic endoscopy allows measurement of the gap in esophageal atresia. Other researchers applied computer tomography scanning for the evaluation of neonates with esophageal atresia [52, 53]. The first measurement of the gap between the esophageal segments is made at the time of initial gastrostomy placement and is repeated at 3-week intervals (Fig. 2). Although some centers have allowed temporary discharge for several weeks on nasopharyngeal suction and gastrostomy feedings [7, 54], the majority of patients with LGEA remain in hospital until delayed primary anastomosis of the esophagus is performed [42, 43, 55, 56]. The parents should be carefully counseled throughout, including necessity and progress of regular gap assessment.

### Delayed primary anastomosis

**Fig. 3:**
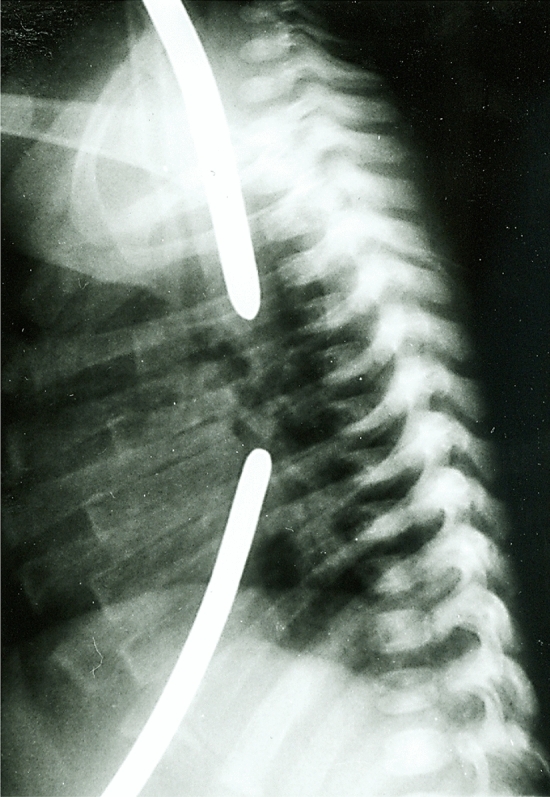
Significant reduced esophageal gap in the same patient at 14 weeks of age

Puri et al. [42] have shown that the maximal spontaneous growth of the two esophageal segments occurs in most patients by 8–12 weeks of age, correlating with doubling of birth weight. By this age, the gap between the two esophageal pouches usually is less than 2 cm and the two ends can be approximated or even overlapped (Fig. 3) [43, 56, 57]. Therefore, it is recommended to perform a delayed primary anastomosis when the patient is about 3–4 months old [58]. Successful primary anastomosis with delays of up to 12 months [54] and initial gaps of up to 7 cm [59] or eight vertebral bodies [60] has been reported. On the other hand, if the gap reduces earlier, and it is felt that careful mobilization will enable the ends to be brought together, definitive repair can be attempted sooner.

**Fig. 4:**
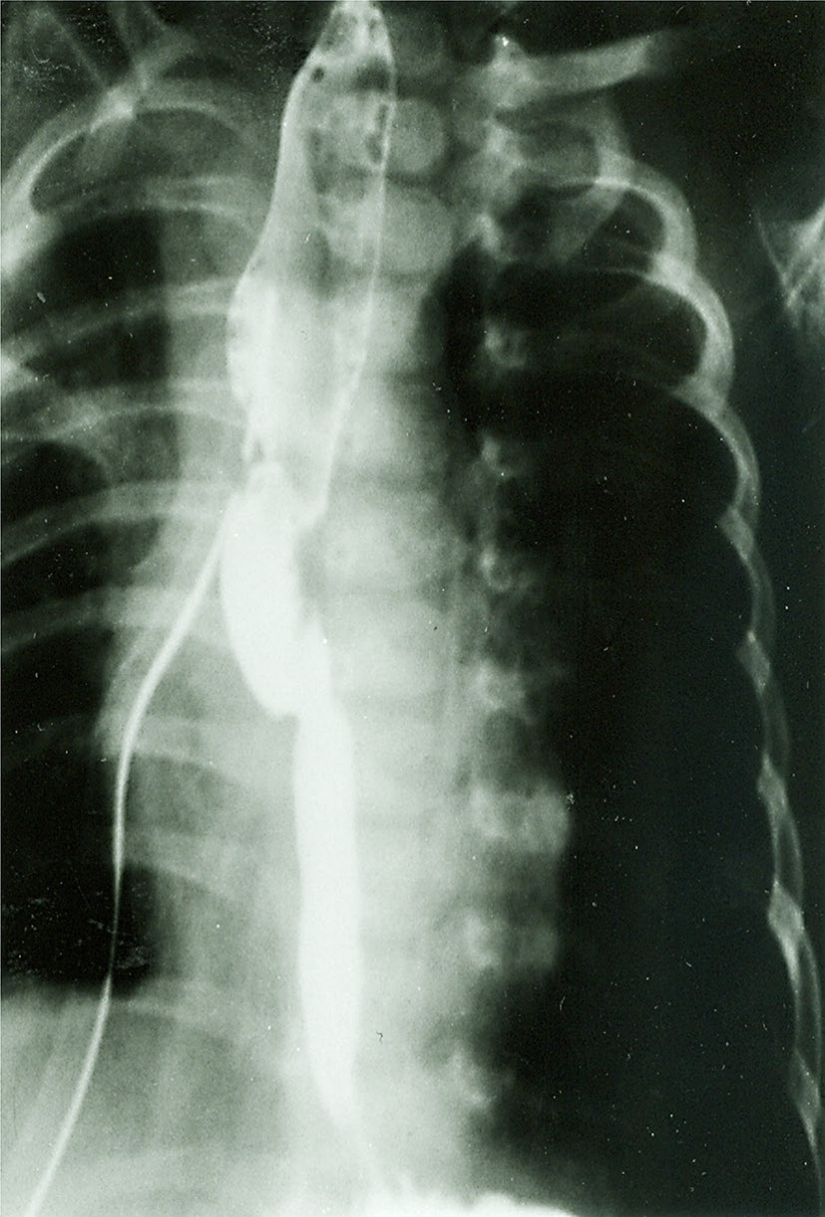
Contrast study on the 10th postoperative day after delayed primary repair showing an intact anastomosis

A preliminary bronchoscopy should be carried out to exclude an upper pouch fistula entering the posterior wall of the trachea, which can be seen in about 20% of cases that presented without intra-abdominal air on initial X-ray [61]. The surgical approach for delayed primary anastomosis of the esophagus in LGEA is similar to that of esophageal atresia repair with tracheoesophageal fistula. In 1999, Lobe and Rothenberg performed the first thoracoscopic reconstruction of esophageal atresia [62]. Currently, the majority of pediatric surgeons still favor the open procedure [33]. In general, this is accomplished through a right posterolateral thoracotomy via the fourth intercostal space using an extrapleural approach. The advantage of the extrapleural approach is that a postoperative anastomotic leak does not contaminate the pleural cavity, requiring prolonged chest tube drainage. By the time of operation, the esophageal pouches appear thickened and hypertrophied [48]. Thus, the tissues have gained strength and are more able to sustain an anastomosis. Dissection and mobilization of the upper esophageal pouch can usually facilitate approximation of the two esophageal segments, enabling an end-to-end anastomosis without excessive tension. Prior to completion of the full-thickness esophagoesophagostomy, a transanastomotic nasogastric tube is inserted. Several investigators have reported using circular myotomy to obtain additional length for the upper pouch [42, 54–56, 59, 63–68]. However, this can lead to damage of the esophageal wall and potentially result in severe stricture, pseudo-diverticulum and extreme dysmotility. A chest drain is not routinely placed as it may increase the rate of anastomotic leakage [69]. Postoperatively, the patient is transferred back to the neonatal intensive care unit, receiving intravenous fluids and antibiotic prophylaxis. Nasopharyngeal suction is continued for the first few days after the procedure. The Replogle tube should be clearly marked to prevent it being accidently passed to the site of the anastomosis, causing damage. If the esophageal anastomosis has been completed under significant tension, the patient is kept electively paralyzed and mechanically ventilated for five postoperative days [70]. Transanastomotic nasogastric feeds can be started on the second or third day following delayed primary anastomosis, and when the infant is swallowing saliva, oral feeds may be commenced. A follow-up contrast study is not routinely performed, but if there is any doubt regarding the integrity of the anastomosis, oral feeds should be withheld and a water-soluble contrast study carried out after 7–10 days postoperatively (Fig. 4). If no leak is present, antibiotics are discontinued, and the patient is allowed to take feeds orally. Furthermore, regular chest physiotherapy is recommended to avoid respiratory infections.

### Potential postoperative complications

In most studies, the survival rate for patients with LGEA after delayed primary anastomosis is reportedly greater than 90% [43, 49, 54, 59, 66–68, 71–78]. Early complications after delayed primary anastomosis are leaks, which may occur in up to 30% of cases [42, 43, 54, 55, 57, 65–68, 72, 75, 78–82]. Fortunately, most anastomotic leaks are minor and will seal spontaneously with antibiotic therapy, nil by mouth and total parenteral nutrition without need for any surgical intervention. However, some researchers have reported major disruption and failure of conservative management with need for drainage or reoperation in up to 15% of their LGEA patients [66, 79]. Anastomotic strictures developed in some series in up to 60% of cases [43, 55, 65, 83]. The presence of a previous anastomotic leak has been found to be the most important risk factor for later stricture formation [48]. Most esophageal strictures respond to periodic dilatations, while only a minority of patients ultimately may require resection and reanastomosis [42, 43, 55, 56, 65, 71, 75, 78, 80, 84]. With meticulous handling of the esophageal ends, preservation of the blood supply and careful inclusion of the mucosa in each and every suture of the anastomosis, strictures can be kept to a minimum [70]. Persistent esophageal strictures are mainly secondary to gastroesophageal reflux [85]. Gastroesophageal reflux that is present following delayed primary anastomosis under tension normally requires a more aggressive approach to treatment. According to most authors [42, 49, 54–57, 59, 63, 65–68, 72, 73, 75, 77–82, 84, 86], up to 30% of their patients, treated by delayed primary anastomosis, require fundoplication in the first year after surgical repair of their LGEA due to either symptomatic gastroesophageal reflux or reflux-associated strictures. Severe esophagitis caused by gastroesophageal reflux occurs only rarely following delayed primary anastomosis and generally can be resolved by fundoplication [63, 78, 82].

### Long-term results

**Fig. 5:**
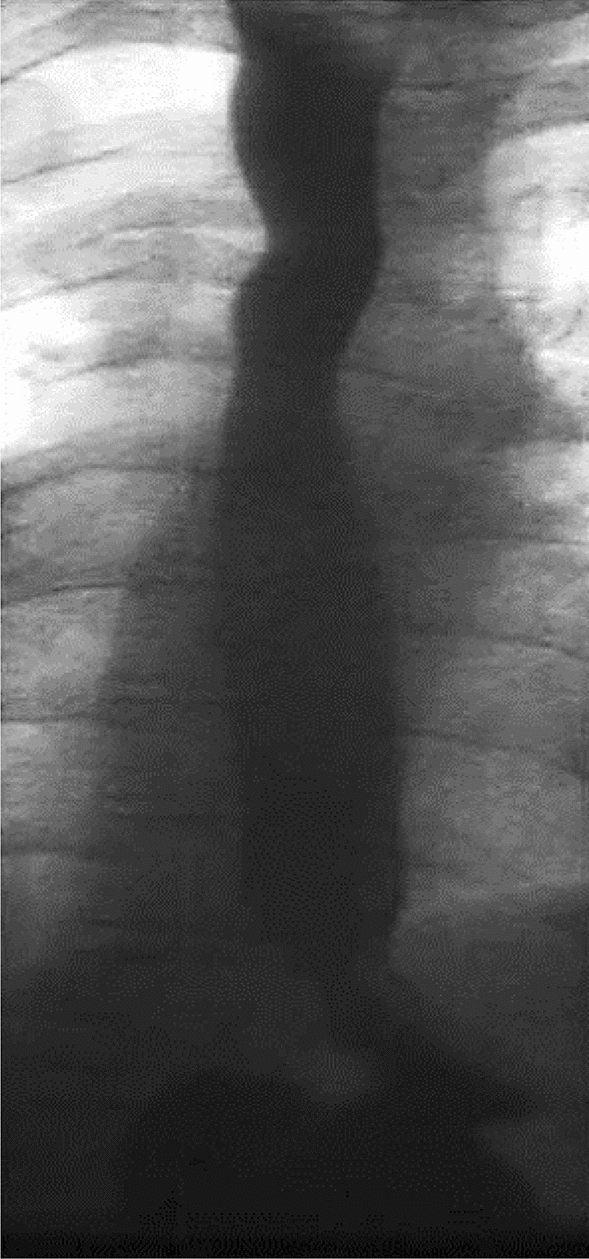
Contrast study in the same patient showing a patent esophagus at 16 years of age

The majority of patients with LGEA who have undergone delayed primary anastomosis are able to eat normally without dysphagia (Fig. 5). Thus, the reported incidence of swallowing difficulties is generally low [43, 64, 68, 80, 86]. LGEA patients who present with dysphagia are often found to have gastroesophageal reflux or reflux-associated strictures on subsequent contrast studies [87]. Recurrent aspiration pneumonia is very uncommon in patients with LGEA who had delayed primary anastomosis but is reported by some authors [57, 63, 64, 86]. Health-related quality of life in children who underwent delayed primary anastomosis seems to be significantly better when compared to their peers who had other types of esophageal reconstruction [88]. Failure to achieve a satisfactory delayed primary anastomosis of the esophagus with need for esophageal replacement is relatively rare and only necessary in very few patients with LGEA [54, 55, 66, 80]. Recently, Lee et al. have indicated that delayed primary anastomosis for LGEA repair has a better long-term outcome compared to esophageal replacement [89]. Most follow-up studies have shown that the majority of patients have normal growth and development curves after delayed primary anastomosis [56, 65, 81, 84]. However, the potential risk of Barrett’s metaplasia and other morbidities highlights the need for continued long-term follow-up in LGEA [56, 78].

## Conclusion and future directions

Delayed primary anastomosis in patients with LGEA generally achieves a favorable postoperative outcome with good long-term functional results. However, the high incidence of gastroesophageal reflux and associated morbidities requires timely intervention to prevent ongoing feeding problems secondary to anastomotic strictures or esophagitis. Long-term follow-up is recommended due to the potential risk of Barrett’s metaplasia. The downside of waiting for the esophageal segments to grow and hypertrophy in LGEA are prolonged hospital stay and constant risk of developing aspiration pneumonia, which requires continuous skilled nursing supervision. It may also be argued that the initial long hospital stay is expensive. These factors must be balanced against reduced long-term morbidity in a child with LGEA that should have a normal life expectancy and against the disadvantages of esophageal replacement [45, 90, 91]. At the moment, there is still no “ideal” substitute for a child’s own esophagus. However, new methods of overcoming the need for esophageal replacement in LGEA are in progress with tissue engineering using acellular or cell-seeded scaffolds to produce a tubular graft that bridges the gap in the continuity of the esophagus [2, 92].
